# Work in progress: power in transformation to postcapitalist work relations in community–supported agriculture

**DOI:** 10.1007/s10460-023-10486-8

**Published:** 2023-07-25

**Authors:** Guilherme Raj, Giuseppe Feola, Hens Runhaar

**Affiliations:** Environmental Governance Research Group, Copernicus Institute of Sustainable Development, Utrecht University, Princetonlaan 8, 3584 CB Utrecht, The Netherlands

**Keywords:** Community-supported agriculture, Agri-food grassroots initiatives, Community economy, Politics of change, Labour

## Abstract

Community-supported agriculture (CSA) initiatives are spaces where diverse work relations are performed. From a postcapitalist perspective, these initiatives attempt to create alternative-capitalist and non-capitalist work relations next to capitalist ones. While analyses of work relations in CSA abound, it remains uncertain how such diversification is made possible and how it is shaped by the micro-politics of and power relations in these initiatives. This paper addresses this gap by analysing how power shapes transformations to postcapitalist work relations in CSA. It provides substantial empirical evidence of multiple manifestations of power enabling or constraining postcapitalist work relations through a comparative case study of three CSA initiatives in Portugal. Results show that while CSA creates postcapitalist work relations that are non-alienated, non-monetised and full of care, they insufficiently unmake unbalanced power relations established in capitalist work relations. This paper argues that, when establishing postcapitalist work relations, the selected CSA initiatives could benefit from actively deconstructing internal hierarchies, de-centralising decision-making power from farm owners and addressing oppressive power relations that are ossified in their local and cultural context.

## Introduction

Community-supported agriculture (CSA) is an agri-food provisioning scheme based on a partnership between the farmer and local consumers where local consumers pre-finance the costs of a harvest season in exchange for a weekly basket of fresh produce from the farm (Galt et al. 2019). Different CSA arrangements exist, and some encompass shared accountability of work duties among the CSA members next to the financial partnership (Feagan & Henderson 2009; Pole & Gray 2013). From a postcapitalist perspective (Gibson-Graham 2006; 2008), CSA initiatives (thereon CSAs) can be viewed as spaces where alternative-capitalist (e.g., in-kind compensation of work) and non-capitalist (e.g., affective compensation of work) work relations exist next to capitalist (e.g., wage labour) ones. The postcapitalist perspective enables us to read the diversity of economic relations and unpack the achievements, contradictions and limitations emerging when CSA attempts to diversify work relations (Vincent & Feola 2020).

Analyses of the types of work relations in CSAs abound. For instance, Nost (2014) compares the advantages and disadvantages of waged, voluntary, and reciprocal work performed in CSAs. Through workshares, CSA members exchange hours of work for a weekly share of the harvest and participate in a non-monetary exchange while also gaining gardening skills (Thompson & Coskuner-Balli 2007; Wilson 2013). Similarly, Watson (2020) argues that CSAs may cease the practice of alienating labour that is deeply inscribed in capitalist work relations. Aspects of non-alienated work performed in CSAs include the remuneration of the labour force by direct and tangible products (not by commodities or wages) that, in turn, encompass a more apparent use value than exchange value. For example, CSA work shareholders produce well-being and public goods in the forms of “food, friendships, exercise, learning, meaningful work, community” (Watson 2020, p. 306). However, CSAs also face the risk of self-exploitation due to a perceived necessity to outcompete agricultural firms through long and intense work shifts in exchange for monetary compensation insufficient to ensure farmers’ well-being (Galt 2013). Additionally, the presence of interns and volunteers at CSA farms may signal solidarity but also the precarity of ecological farming as a viable and rentable agricultural venture (Ekers 2019).

While accounts of work relations in CSA abound, it remains uncertain how this diverse configuration of work relations is made possible. Particularly, it is unclear how farm owners and CSA members negotiate the creation and perpetuation of postcapitalist work relations and for the benefit of whom; how the farm infrastructure influences the constitution and diversification of work relations; and whether CSA members tackle culturally institutionalised practices and discourses that hinder postcapitalist work relations. Scholars interested in work relations in CSAs have considered questions of power. They have shown, for instance, that CSAs implement democratic governance structures allowing members to influence decisions and define work distribution (Watson 2020) and prioritise autonomy over rules in the work arrangement between farm owners and apprentices (White 2013). Others have shown that CSAs face several obstacles posed by policies and procedures to access land established in modern capitalist societies when creating alternative work relations (e.g., Galt 2013; Ekers 2019). This paper builds on these findings to further advance the understanding of how power relations shape the accomplishments and difficulties of CSAs to create and perpetuate diversified work relations while offering practical insights into transformations beyond capitalism in CSAs. Destructive modes of interaction with the social and natural environment are not simply a remediable side effect but rather a characterising trait of modern capitalist societies; thereby, challenging them is a fundamental endeavour for sustainability transformations (Feola 2020; Feola et al. 2021).

This paper addresses recent calls for further theorisations of power that engage critically with the analysis of forms of power relations underlying issues of inequality and injustice in postcapitalist transformations (Gabriel & Sarmiento 2020). It aligns with recent research that has emphasised the need for a deeper examination of questions of power in postcapitalist formations in agri-food systems. For example, Turker and Murphy (2021) and Morrow and Davies (2022) examined individual and collective power in agri-food grassroots initiatives to establish postcapitalist agri-food practices and Wilson and Mutersbaugh (2020) investigated conflicts between agriculture cooperatives and certification companies in attempts to forge postcapitalist futures. Drawing on the foundational work of Gibson-Graham (2006, 2008), this paper views transformations toward postcapitalist work relations as a political process of diversification that reattributes value to alternative- and non-capitalist work relations traditionally undervalued and invisibilised. To analyse how power shapes transformations to postcapitalist work relations, this paper employs a relational and multidimensional typology of power that includes human and non-human agency and historical and situated processes of constitution of agency and power relations (Allen 2021). We combine those theorisations of power with the approach of Feola (2019) and Feola et al. (2021), who consider transformations as processes of unmaking capitalist relationships and practices that make space for the emergence of postcapitalist alternatives. We focus on the transformation of three aspects of work relations discussed in the CSA literature: alienation, monetisation, and care.

Three CSAs in Alentejo, South Portugal, serve as case studies. They are led by the farm owners, yet each one employs different levels of horizontal organisation. These cases provide a comparative ground to analyse how different power arrangements shape the achievements, contradictions and limitations of transformations towards postcapitalist work relations. The experiences of these three CSAs are inherently shaped by their regional dominant agri-food system that has been the main stage of the agrarian modernisation of Portuguese agriculture (Calvário 2022). This paper contributes to research on agri-food grassroots initiatives for transformations to sustainability beyond capitalism by uncovering the processes through which postcapitalist transformations unfold in these three initiatives.

## Theoretical background

### Postcapitalist work relations

Postcapitalist analyses of diverse and community economies have formed the basis for studies of social transformations beyond capitalism in agri-food systems (Harris 2009; Trauger & Passidomo 2012; Vincent & Feola 2020; Moragues-Faus et al. 2020; Rosol 2020; Morrow & Davies 2022; Sharp et al. 2022). A specific line of research has focused on work relations as a crucial aspect of postcapitalist agri-food system transformation. In particular, empirical studies of CSA, without using the terms and frameworks of postcapitalism systematically, have shown that these initiatives create diverse work relations that combine capitalist, alternative-capitalist, and non-capitalist work^1^ at different phases of their operations (e.g., Cone & Myhre 2000; Galt 2013; Wilson 2013; Nost 2014; Vincent & Feola 2020; Watson 2020; Cristiano 2021). The creation of diversified work relations in CSAs can be understood as an endeavour towards a postcapitalist arrangement of work relations in agri-food systems.

This study focuses on three interconnected aspects of work relations discussed in the CSA literature, and agri-food grassroots initiatives for transformations to sustainability more broadly: alienation, monetisation, and care. They are relevant areas of investigation to analyse the achievements, contradictions and limitations of CSA attempts to diversify work relations. Furthermore, they offer critical insights to inform our analysis of how attempts to create work relations that are non-alienated, non-monetised and full of care address issues of social injustice, environmental harm, and natural resource exploitation underlying capitalist work relations (e.g., Jarosz 2011; Galt 2013; Wilson 2013; Watson 2020).

#### Alienation

The Marxist definition of alienation of work within capitalism comprises four key dimensions: (1) alienation from the product of labour, (2) alienation from the process of labour, (3) alienation from other workers, and (4) alienation from human potential (Marx 1959 cited in Watson 2020). In the context of agri-food systems, alienation results from the capitalist organisation of agri-food relations that depletes the use value of food and, in turn, imbues food with exchange value used for commodity trading and capital accumulation in market operations. Because the commodification of food has historically implied less favourable wages and benefits for workers along the supply chain, alternatives to capitalist organisations of agri-food relations must acknowledge and address workers’ struggles (Minkoff-Zern 2017).

Different examples of how CSAs address alienation when organising work relations include work performed by CSA members for a clearly defined purpose and outcome that they can directly enjoy (e.g., food); farm work that generates deeper connections between humans, other species, and the natural environment; and community work that provides a social support network for members (Watson 2020). Also, in CSAs, farmers and co-producers experience excitement when working in the fields and discovering the practicalities of food production alienated by the capitalist separation of food production and consumption (Thompson & Coskuner-Balli 2007). However, it is crucial to notice that capitalist relations of production dependent on wage labour may still exist in parallel to non-alienated work in CSAs, and waged work does not necessarily alienate workers (e.g., cooperatively defined wage) (Watson 2020). Additionally, non-alienated work relations in CSAs may only partially signal transformation if these initiatives do not problematise the labour-intensive character of ecological farming and the need to promote and protect the well-being and benefits of workers (Minkoff-Zern 2017). The maintenance and diffusion of non-alienated work relations is partially a result of the prefigurative capacity of these initiatives and also depend on their capacity to confront the capitalist labour regime in agri-food systems (Myers & Sbicca 2015).

#### Monetisation

In the capitalist organisation of work, monetised work relations (e.g., paid and socially recognised work that produces commodities and services) receive more appreciation than non-monetised work relations (e.g., unpaid work that produces well-being) (Dengler & Strunk 2018). Historically, the capitalist organisation of agri-food relations resulted in the increasing professionalisation of on-farm labour; yet, non-monetised work performed by family members and intermittent apprentices persist and can be understood as part of a broader negotiation of the “agrarian question”, or the strategies employed by small-size farms to exist in the face of an expanding capitalist-led industrialised agri-food system (Ekers et al. 2016).

Different forms of non-monetised work relations in CSAs include workshare membership, which entails volunteering work for farming and distributing activities in exchange for a weekly vegetable basket (Thompson & Coskuner-Balli 2007; Wilson 2013). Also, additional voluntary work by CSA members and externals is offered as free input (Cristiano 2021); for instance, volunteers and interns are temporarily employed to support farming work in exchange for access to agricultural knowledge, food, and shelter (Galt 2013; Ekers 2019). However, non-monetised work relations may cause work precarity in CSA farms, as evidence shows cases of self-exploration (Galt 2013) and economic fragility (Ekers 2019) in CSAs. Ekers et al.(2016) argue that the reliance of ecologically-oriented farms, like CSA farms, on interns, apprentices and volunteers to ensure their economic and ecological viability is inherently contradictory. It simultaneously signals (i) economic precarity of these farms vis-à-vis the competitive pressures created by the capitalist agri-food system and (ii) non-economic and moral motives to associate these forms of non-monetised work with their ethical and political engagements. These moral motives may also normalise precarious work conditions, instead of encouraging the active contestation of structural conditions that obscure the importance of wages, insurance coverage and other benefits for interns, apprentices and volunteers (Weiler et al. 2016). In line with Sbicca (2015) and Levkoe and Offeh-Gyimah (2020) the presence of precarious working conditions of interns, apprentices or volunteers in CSAs that are justified by moral motives also relates to questions of class privilege and to activist or an unprotected worker subjectivities in these initiatives.

#### Care

In Western capitalist societies, reproductive work, such as care work performed to regenerate social and ecological lives, is understood as a “maintenance basis” for productive work, for example, food provisioning work performed to produce exchange value and generate an income (Dengler & Strunk 2018). While the latter gains visibility and recognition in the public sphere (e.g., work legislations), the former is visible and recognised only in the private sphere (e.g., internal organisation of the household). Historically, the invisibility of reproductive work in the public sphere of Western patriarchal societies has also reinforced gender inequality (Duffy 2007). Besides this traditional conceptualisation of interhuman and social relations of care, debates on transformations to sustainable agri-food systems have discussed socio-ecological notions of care and stewardship in connection to the soil, the land and natural resources (Jarosz 2011; Pungas 2020).

Studies have provided evidence of how CSAs value work relations full of care (Delind & Fergunson 1999; Cone & Myhre 2000; Wells & Gradwell 2001; Jarosz 2011). Beyond food production and delivery, CSA members, particularly women, work for the maintenance of the community by building a sufficiently large, committed, and stable membership (Cone & Myhre 2000). CSAs characterise their resource management, food production, and other ecosystem interactions by employing care motives and practices (Wells & Gradwell 2001; Jarosz 2011). Yet, it remains a challenge to negotiate the valorisation of caring work relations and practices over productivist imperatives as CSA’s economic and ecological viability are mainly associated with the latter approach to farm labour (Jarosz 2011). Although CSAs do not deliberately challenge or alter structures of oppression that result in gender discrimination, they may create social spaces for women’s self-identification and reproductive roles, including care practices such as community building (Delind & Ferguson 1999).

### Power and postcapitalist transformations

We adopt the typology of relational power conceived by Allen (2021) that provides a typology of three distinct approaches to power: *action-theoretical*, *constitutive*, and *systemic* power. This multidimensional and relational conceptualisation of power deviates from understanding power as something “owned” and exercised by agents independently of its embedded context, implying a static manifestation of power incompatible with the changing dynamics inherent to transformation processes (Ahlborg 2017; Raj et al. 2022). In Allen’s typology, *action-theoretical power* is related exclusively to the realm of human agency. Its focus is two-fold: the *intentions* of those who exercise power towards others and the surrounding environment (e.g., the exercise of power-to act or refrain from action and the power-over others) and the *dispositional abilities*, or the human attributes, that are unequally distributed in society and may be exercised (e.g., decision-making power at the disposal of elite actors). *Constitutive power* expands agency to non-human elements and refers to power emerging from the relations between human and non-human actors (e.g., the hammer in a worker’s hands). *Systemic power* accounts for the historical and situated processes that result in culturally institutionalised practices, legal institutions, and discourses that enable some human and non-human agents to exercise power over others or engender abilities in some actors but not others (e.g., energy distribution infrastructure stabilises socio-economic inequalities).

We conceptualise transformations as a “multilevel (individual, social, socio-ecological) and multidimensional (temporal, spatial, symbolic, and material) range of situated processes that can be used strategically to make space for sustainable alternatives” (Feola 2019, p. 992). Such a perspective is relevant since societal transformation towards sustainability “necessarily rests on challenging and transforming capitalist institutions, and their cultural, social and political architecture” (Feola 2019, p. 978). Rather than conceptualising transformations as a process of mere addition and innovation of supposedly sustainable socio-technical solutions, values or practices, Feola et al. (2021) posits that more research is needed to examine how processes of unmaking unsustainable capitalist relationships and practices are possible conditions for transformations. Feola et al. (2021) introduced an inventory of possible processes of unmaking discussed across the social sciences, as shown in Appendix A. Previous work based on the concept of unmaking capitalism has been used to explore the construction of postcapitalist realities in a Colombian peasant movement (Feola et al. 2021) and the role of unlearning in the conversion to solidarity-payment schemes in two Dutch CSAs (van Oers et al. 2023).

These relational and political perspectives on power and transformations are employed in this paper to analyse how CSAs reattribute value to non-alienated, non-monetized and caring work relations. Different processes of unmaking capitalism may be a pre-condition for the revaluation of these three forms of alternative- and non-capitalist work relations. Based on similar experiences of unmaking discussed in the postcapitalist literature and studies on work relations in CSA, we select six concrete processes of unmaking capitalism: unlearning, sacrifice, everyday resistance, resistance, refusal and defamiliarisation (Feola et al. 2021). In particular, we examine how different power relations between CSA members, between them and non-human actors influenced by the regional and historical context, enable or constrain this transformation and the revaluation of work relations.

Table 1 shows how we operationalised the typology of power to six concrete processes of unmaking capitalism in transformations to postcapitalist work relations in CSA. The first column introduces the core idea of the selected process of unmaking. Then, the table cells of the remaining three columns illustrate how the three types of power could shape each process of unmaking in the context of work relations. The illustrative examples are based on similar experiences of unmaking discussed in the literature of work relations in CSAs and postcapitalist transformations.

**Table 1:** Power in processes of unmaking capitalism (adapted from Allen [2021] and Feola et al. [2021])

Processes of unmaking (core idea)	Types of power		
	Action-theoretical	Constitutive	Systemic
Unlearning (Consciously letting go of old values, norms, or beliefs)	Farm owners consciously let go of exploitative work routines	Farmers let go of old farm infrastructure that generates exploitative labour routines	Access to education defines who can critically reflect and let go of old exploitative working routines
Sacrifice (Giving up something for something else of higher value)	Consumers give up self-interest to do voluntary work for the community	Rotating shift schedules supports consumers to give up self-interest to do voluntary work for the community	Class privilege defines who can give up self-interest to do voluntary work for the community
Everyday resistance (Covert acts of opposition to abusive or oppressive power relations)	Farm employees act covertly to erode the legitimacy of the “boss”	Farm employees use heavy farming tools to slow down manual work (purposeful inefficiency)	The culturally institutionalised “boss–worker” relations foster farm employees to oppose hierarchy covertly
Resistance (Overt acts of opposition to abusive or oppressive power relations)	Farm owners act overtly to oppose environmentally harmful working relations	Farm owners oppose the adoption of agro-chemical inputs to avoid harming the soil and employees	Dominant discourse about the economic profit of agro-chemicals supports farm owners to organise protests
Refusal (Rejection of an imposed definition of a situation, subjectivity, or social relation)	Farm employees reject subaltern identities imposed by authoritarian figures	Farm employees reject the usage of certain work tools associated with subaltern identities	Gender norms determine different abilities in men, women, or non-binary to resist oppression
Defamiliarisation (Removal of an object from the sphere of automatised perception)	Consumers become dishabituated of shared understandings of the purpose of work	The dishabituation of industrial meanings attributed to food that hinder consumers to perform farm work	Access to education defines who can critically reflect and decide to become dishabituated of shared understandings of work

## Material and methods

### Case studies

This study adopted a comparative case study approach. Comparing the similarities, differences, and patterns across various CSAs enabled us to document and analyse a multitude of power manifestations influencing the creation, consolidation, and perpetuation of work relations that are non-alienated, non-monetised, and full of care.

Three CSAs in Portugal served as case studies (Table 2). For the sake of anonymisation, we refer to each selected initiative by different codes: CSA1, CSA2, and CSA3. We used the Portuguese CSA network platform to choose the case studies as the network offered a list of initiatives active for a minimum of two years. We expected work relations to change over time and selected initiatives that existed for multiple years. Transformations towards postcapitalist work relations in these CSAs have a tentative and incomplete nature and are currently in progress.

**Table 2:** Characteristics of the three cases of farmer-led CSAs in Portugal

	Case studies		
	CSA1	CSA2	CSA3
*History*			
Farmland acquisition	Farm owners’ inheritance	Farm owners’ inheritance	Land bought from own savings
Start of farm operations	1990	2009	2017
Start of CSA operations	2015	2019	2019
*Membership*			
Number of farm owners (fall 2021)	1	2	2
Number of employees (fall 2021)	35	–	2
Number of co-producers (fall 2021)	160	24	26
*Food production*			
Farm size (HA)	600	3.4	2
Farm activity	Horticulture and Livestock	Horticulture	Horticulture
Approach to agriculture	Agroecology	Agroecology	Agroecology
Labour	Employees	Farm owners	Farm owners; employees; co-producers
*CSA administration*			
Logistics	Farm owners; employees; co-producers (intermittently)	Farm owners; co-producers	Farm owners; employees; co-producers
*Organisation*			
Level of horizontality	Low	Medium	Medium–High

We selected three farms that have been converted to CSAs by their owners. We refer to these initiatives as farmer-led CSAs. We acknowledge that the results of this study are shaped by the micro-politics of this specific type of CSA, which may differ from other types of CSAs, such as consumer- or cooperative-led CSAs. While the three cases were farmers-led, each employed different levels of horizontality in their internal decision-making processes and distribution of work tasks and responsibilities. By levels of horizontality, we refer to the degree to which decision-making and work duties were organised through participatory means and employed shared work accountability among all members.

We distinguish among three general types of members across the three CSAs, as identified by CSA members themselves. *Farm owners* are the owners and main inhabitants of the farmland who manage and execute farm activities. *Co-producers*^2^ are the local consumers who pre-finance the costs of a harvest season, receive fresh produce weekly, and can participate in decision-making and work activities organised by the CSA. *Employees* are the waged workers performing food production or administrative work under temporary, part-time, or full-time contracts. The farm owners across the three cases are new entrants. In CSA1 and CSA3, the co-producers are primarily urban dwellers, while in CSA2, they are mainly neo-rurals. The employees in CSA1 have a range of agriculture skills, from semi-skilled to highly skilled, and they are predominantly from rural areas, with a few from urban backgrounds. In CSA3, the employees are mainly semi-skilled in agriculture and live both in rural and urban areas. In contrast, CSA2 operates without any employees. Furthermore, the payment scheme varies across the three cases: in CSA1, co-producers can choose between monthly or semi-annual payments, whereas in CSA2 and CSA3, co-producers make monthly payments.

The three CSAs are located in Alentejo, South Portugal. Historically, this region has played a significant role in the modernisation of the Portuguese agri-food sector (Calvário 2022) and is currently characterised by extensive monoculture and greenhouse farms that primarily cultivate olives, berries and other commodities (INE 2021). While the rural population is declining and aging (INE 2022), a growing neo-rural population has been formed mainly by immigrants from other European urban centres seeking a lifestyle change (Esteves 2017; Novikova 2021) and from south Asian countries looking for work opportunities in farms and greenhouses to fulfil their social aspirations and economic necessities (Pereira et al. 2021).

### Data collection

Data on the three case studies were collected through desk research and fieldwork conducted between April and November 2021. Through participant observation, we gained a better grasp of the work relations and farm operations singular to each case and a more in-depth understanding of power relations between members of the CSA and between them and the farm infrastructure. Participant observation was carried out by the first author who visited the three farms for two to four weeks between June and November 2021 and participated in daily working routines at the farm, delivery of CSA baskets, and CSA meetings and assemblies (online and in-person). During the farm visits, semi-structured interviews were conducted with 36 CSA members (at least 10 members of each CSA). The first people interviewed in each initiative were the farm owners, who had an overall view of the different farm operations and CSA members. The co-producers and employees interviewed were selected through snowball sampling. Interviews were conducted in Portuguese, the mother tongue of both interviewer and participants. Topics covered in the semi-structured interviews included the motivation and objectives of farm owners, co-producers, and employees to work for the CSA; the explanation of their different tasks, responsibilities, and roles and how they related to those of other members of the CSA; the explanation of how decisions are made and who participates in them; and the achievements and difficulties to foster the participation of different CSA members.

### Data analysis

We used the conceptual framework presented in Table 1 for coding the interviews, internal documents of the CSAs, and fieldwork notes. Coding enabled us to identify instances of unmaking capitalism entangled in the making of postcapitalist work relations and how they were shaped by different manifestations of power in the reconstructed transformation in each case study. We then organised the findings based on three work relations aspects: alienation, monetisation, and care. While the identification and choice of these aspects were informed by a literature review of work relations in CSA, their relevance for this study emerged from the empirical investigation of the specific case studies. In the final stage, we used the conceptual framework (Table 1) to contrast the results across the case studies, which led to further insights and suggested further explanations of how power enabled or disabled (un)making in the CSA’s attempts to diversify work relations.

## Results: power in the unmaking of capitalist work relations for making postcapitalist ones

By and large, the creation of the three CSAs diversified work relations at the farms. These initiatives negotiated to different extents alternatives to capitalist relationships and practices in their internal work arrangement. While their attempts to revalue work relations that were non-alienated, non-monetised and caring were successful at times, they also faced barriers in their endeavour and reproduced aspects of capitalist work relations, such as hierarchal organisation, self-exploitation and discrimination.

In the three cases, the CSA fostered the participation of CSA members in decision-making, logistics, and food provisioning operations, and it created new tasks and responsibilities (e.g., community building, organisation of assemblies, coordination of distribution points) and new kinds of worker subjectivities. In terms of non-alienated work relations, the reoccurrence of CSA assemblies, help-out gatherings, and informal events across the three cases factored in the de-alienation of co-producers and employees by involving them in and increasing their awareness of farm operations. While in CSA3 we observed progressive accountability of co-producers and employees over the CSA, in CSA1 and CSA2 such accountability remained limited. Particularly, hierarchal interactions between farm owners and employees, co-producers and volunteers hindered the creation of non-alienated work relations. Concerning non-monetised work relations, CSA2 and CSA3 mainly relied on non-monetised work performed by temporary volunteers and co-producers. In contrast, CSA1 expanded the number of monetised work relations performed by salaried employees to manage production and logistic operations. The involvement of co-producers and volunteers with unpaid work was entangled with class privilege and simultaneously signalled the economic fragility of these initiatives, with the exception of CSA1 who afforded salaried employees. Regarding work relations full of care, the three CSAs reinforced the financial viability of ecological farming operations and increased the visibility of care work traditionally invisibilised. Yet, all three cases struggled to resist culturally institutionalised practices that devalued care work, which in some cases also resulted in gender discrimination.

Table 3 summarises the main aggregate findings concerning how power enabled or constrained processes of unmaking capitalism entangled in making work relations that are non-alienated, non-monetised, and full of care across the three CSAs. The concrete cases of unmaking identified refer to particular moments when CSA members individually or collectively faced a barrier to implementing their alternative- and non-capitalist work relations and saw the need to rethink or abandon established capitalist relationships and practices. In the remainder of this section, we present these results in detail.

**Table 3:** Power shaping the unmaking of capitalism in the making of work relations that are non-alienated, non-monetised, and full of care

Entanglement of unmaking capitalism in the making of postcapitalist work relations	Power	
	Enabling the unmaking of capitalist work relations	Constraining the unmaking of capitalist work relations
Unmaking the alienation of work for making non-alienated work relations	• Farm owner exercised power to let go of the productivist paradigm and create meaningful and enjoyable work relations • The synergetic relations between workers and the farm infrastructure constituted meaningful and enjoyable work relations • CSA members gave up leisure time to work for the CSA • Farm owners exercised power to decentralise tasks and responsibilities and empowered co-producers to take accountability for CSA operations • Co-producers and employees created pejorative terms and refused to participate in meetings under the terms defined by the farm owner to implicitly undermine his power over them • CSA members exercised collective power to resist hierarchical work relations among farm owners, employees, and CSA members	• Farm owners centralised decision-making power to distribute tasks and responsibilities among CSA members • The historical and situated constitution of boss–worker relations in the agricultural sector reinforced the centralisation of decision-making power on farm owners
Unmaking the monetisation of work for making non-monetised work relations	• Co-producers sacrificed individual preferences to pursue the collective responsibility of farm owners’ well-being • Co-producers refused monetary compensation for their voluntary work, which helped transform hierarchical work relations into collaborative ones • CSAs enabled the creation of new producer and consumer subjectivities and empowered CSA members to discard the service mentality	• The refusal of monetary compensation for work performed for the CSA was entangled with class privilege • Acting upon the new producer subjectivities in CSAs was a contradictory experience that demotivated producers to pursue collaborations with co-producers
Unmaking the structural separation of productive and reproductive labour for making work relations full of care	• The negotiations over the name and meaning of the CSA basket de-automatised taken-for-granted perceptions and enabled the creation of new meanings to interactions in the CSA • Farm owner exercised power to foster collective responsibility for farm infrastructure. CSA members inscribed meaning into farm infrastructure that fostered collective action • Rotating schedules conceived by CSA members partially constituted hindrance to the devaluation and invisibility of care work • Female farm owner exercised power to resist both visible and invisible gender inequality associated with gender division of work	• Devaluation of care work deeply inscribed in the local culture hindered collective accountability of housekeeping tasks. Only some CSA members sacrificed personal preferences to perform care work for the community • Gender norms shaped the division of work and distribution of value across productive and reproductive work

### Unmaking the alienation of work for making non-alienated work relations

Results confirm Watson’s (2020) claim that CSAs counter the alienation of labour as members work to produce outcomes that contribute to the well-being of members and the farm’s ecosystem. Firstly, co-producers who frequently participated in farm activities expanded their awareness of the practicalities and challenges of producing and distributing food. Such an awareness, in turn, enabled co-producers with limited experience in agricultural production to build an affirmative attitude in decision-making meetings, as pointed out by one co-producer:

I became aware that the croutons were handmade and that it was a job that didn’t pay off […]. When someone makes a proposal [during decision-making meetings], one is aware of this sort of practical information, right? […] Participation becomes more conscious. (co-producer, CSA1)

Secondly, work relations that produce outcomes directly benefiting CSA members included co-producers who worked voluntarily in the field to help grow the food they consumed and co-producers who took on unpaid administrative activities for the CSA. One co-producer explained their motivation to take on the responsibility of creating newsletters for the CSA members:

I met many interesting people in the CSA who became my friends! I met, for example, a person with whom I’m working now [...] I think I can make a small contribution like this [production of the CSA newsletter] to maintain and make this community flourish, let’s say, to bring more interesting people into it. (co-producer, CSA2)

Thirdly, CSA farm owners and co-producers strengthened their social ties during work activities. One co-producer commented on the importance of organising shared meals during the help-out gatherings at the farm:

The mealtime is a time for conviviality, and it is a time that is part of the whole working day as a community. There is more fraternisation, and this part of social involvement is closely linked to the concept and the objectives of the CSA. Hence the importance of mealtime being greater here than in a traditional job, or in traditional ways of working in offices or industries. (co-producer, CSA3)

As this brief overview clearly shows, some work relations at the three CSAs included a level of non-alienation. These results need to be interpreted with caution, as creating non-alienated work relations in CSAs is not a comprehensive process and alienation may still exist in different levels of their internal work arrangement. When trying to create non-alienated work relations, different capitalist relationships and practices were actively unmade. We highlight four concrete processes of unmaking capitalism identified in the three CSAs, as shown in Table 4. We discuss them in turn.

**Table 4:** Evidence of unmaking processes in the making of non-alienated work relations across the three CSAs

Non-alienated work relations	Process of unmaking	Case		
		CSA1	CSA2	CSA3
Awareness of the activity of labour itself	Unlearning	–	–	Farm owner rejected the productivist paradigm
Production of outcomes that directly benefit the CSA members	Sacrifice	Co-producers and farm owners give up leisure time to work for the CSA	Co-producers and farm owners give up leisure time to work for the CSA	Co-producers and farm owners give up leisure time to work for the CSA
Work relations that strengthen social ties	Resistance and everyday resistance	Co-producers and employees implicitly opposed the farm’s hierarchy	–	Co-producers object to the centralisation of decision-making power on farm owners

**Unlearning:** Unlearning refers to the conscious decision not to act or think in “old” ways (Appendix A). One farm owner of CSA3 rejected the dominant productivist arrangement of the farm infrastructure that prioritised high yields by reinforcing exploitative work routines. The deliberate rejection of productivity as the main driver for arranging farm infrastructure and farm work enabled the creation of enjoyable work routines. The farm owner decided to no longer arrange the horticulture beds in ways that required unpleasant positions and long working shifts. In doing so, the farm owner expected co-producers to enjoy their voluntary work at the farm, thereby increasing their participation in farming activities. As explained by the farm owner, they noticed that co-producers with limited farming experience worked less comfortably in the field when the size of the horticulture beds prioritised space for growing crops instead of room for people to work:

With corridors of 50 cm, the crops can grow, but the corridors are very narrow, and it’s challenging for someone to be there. With more than one-metre corridors, it is enough for people to weed, even lying down, without feeling too uncomfortable. So I started to change that [working] dynamic a bit as a consequence of what I observed. People who are not used to working on the land often revealed strategies to me, and I also realised in them what bothered me. […] It is not only the productive nature [of farming] that matters, but the social nature of making people feel good when they come to work in the field. (farm owner, CSA3)

From a standpoint that intersects *action-theoretical power* and *constitutive power*, the power of the farm owner to create enjoyable work relations and encourage co-producers’ participation in farm activities was expanded by a type of farm infrastructure that prioritised synergetic interactions between co-producers and the horticulture beds instead of productivity and exploitative work routines.

**Sacrifice:** Acts of sacrifice entail a solid moral component that prioritises long-term benefits for the community over short-term individual benefits (Appendix A). Farm owners and co-producers across the three CSAs gave up individual self-interest to prioritise work that benefited the CSA. Acts of sacrifice were identified more frequently in CSA2 and CSA3 than in CSA1. Arguably, this might be the case because, in CSA1, most of the CSA operations were executed by the farm employees, which made CSA1 less dependent on co-producers than in the other two cases. In CSA2 and CSA3, co-producers gave up their leisure time to participate in CSA activities. One co-producer of CSA3 explained their motivation to join in help-out gatherings during the weekend, despite feeling tired from other working activities:

One day at the field, and I get body aches. [The farm owners] might get even more body aches, as they work in the field every day. So I do think it is good that there is at least one day [help-out gatherings] that we [co-producers] are there to support them. (co-producer, CSA3)

Similarly, farm owners gave up their leisure time to work for the CSA. As explained by one farm owner of CSA2:

[Before the creation of the CSA] we were always working. There was not much difference between weekdays and weekends. In reality, today is the weekend, and we are working too. (farm owner, CSA2)

As initiators of CSAs, farm owners envisioned a horizontal organisation of CSA operations and shared responsibility with co-producers to cope with the risks and benefits of agriculture. However, the degree of participation of co-producers in the organisation and operations of the CSA often fluctuated, creating internal organisation challenges. For instance, farm owners of CSA3 had to continuously hold co-producers accountable for their commitment to distributing the vegetable boxes one week per month. Farm owners of CSA2 often reminded co-producers to clean and organise the distribution point after collecting their vegetable baskets. One co-producer of CSA2 viewed the additional work performed by farm owners as beneficial for the collective:

[The farm owner] organises activities for co-producers to help out with farm work and social events for everyone to discuss current topics.[…] I see that [the farm owners], who are the main drivers [of the CSA], do things beyond what they should do. (co-producer, CSA2)

From an *action-theoretical power* perspective, these findings indicate that CSAs relied on the *ability* of farm owners and co-producers to give up leisure time to work for the CSA. However, different motives influenced farm owners’ and co-producers’ sacrifice, also in relation to class privilege. While moral and solidarity motives underlay co-producers’ sacrifice, and their involvement in CSA work was optional, farm owners financially depended on the CSA and gave up expected leisure time on weekends to meet the production demand. Additionally, the viability of CSAs depended on farm owners’ *ability* to coordinate the decentralisation of and co-producers’ commitment to tasks and responsibilities to achieve their promises of horizontal organisation and co-responsibility.

**Resistance and everyday resistance:** Resistance is an overt, intentional action that opposes structures of domination (Appendix A). Co-producers in CSA3 resisted through visible acts the centralisation of decision-making power on farm owners to strengthen just and collaborative relations in the CSA. Co-producers objected to the internal division of tasks and responsibilities that allocated a coordination role and greater decision-making power to farm owners to claim decision-making power to co-producers. While some co-producers accepted farm owners’ coordination, others were critical of the hierarchal interactions that such a coordination role imposed on the CSA:

Within a CSA, the centrality is in the peasants who make your food, but at the same time you want a community that supports them. There’s centrality and a hierarchy, in some way, even though this centrality is not wanted. […] My point is: centrality is hierarchy. In other words, who makes the decisions for the group is not the group. (co-producer, CSA3)

During a help-out gathering in October 2021, CSA3 co-producers voiced concerns about the uneven distribution of work tasks and responsibilities coupled with the centralisation of decision-making power on farm owners. Subsequently, CSA members organised a mapping exercise to identify the different tasks in the CSA and to whom they were assigned with the aim to reconfigure task division and allocate more decision-making power to co-producers. For instance, the following CSA assembly in March 2022 was the first one prepared and facilitated by co-producers and not the farm owner.

In contrast, everyday resistance refers to disguised, seemingly invisible acts of opposition to abusive power (Appendix A). Co-producers and employees of CSA1 covertly resisted the centralisation of decision-making power on the farm owner. In particular, co-producers and employees commented on tactics to resist the centralisation of power performed during meetings. One employee commented that the farm owner implemented sociocracy techniques to facilitate team meetings without previously consulting employees. Although the employee did not fully grasp the format and the purpose of sociocracy and felt demotivated to participate, they attended the meetings fearing possible remarks from the farm owner about their absence. The employee commented that they purposefully did not speak nor contribute to the conversations as a tactic to discreetly show discontent and opposition to the team meetings. Similarly, one co-producer commented that, together with other co-producers, they referred to the farm owner’s participation in CSA meetings as “[name of the farm owner]splaining”, or a type of condescending explanation of agenda points, in an attempt to undermine the legitimacy and authority of the farm owner.

When interrogated about the decentralisation dynamics in their CSA, interviewees of CSA1 articulated a historical constitution of the work culture in Portugal that perpetuates a hierarchical relation between land owners and farm workers.

[The workers’ cooperative meeting] was long, and [the farm owner] spoke the most. I feel that he doesn’t want it to be that way. I feel he doesn’t want to be the land owner and the boss. The person that people see in this position of hierarchy, right? […] Fortunately, he tries to employ people in the area and is creating jobs for locals, which is great. However, what you get there is the culture of local people, especially the older generations, which is very worker–boss oriented. (employee, CSA1)

Two considerations of the role of power in (everyday) resistance can be made upon the instances mentioned above. Firstly, in *action-theoretical power* terms, achieving a participatory and horizontal organisation of work relations in the CSA relied on the farm owners’ *ability* to decentralise power. However, decision-making power remained *centralised* on farm owners. Additionally, in systemic power terms, the constitution of work relations between farm owners and farm workers was influenced by a historical and situated process that allocated more decision-making abilities to farm owners than farm employees. Secondly, through a perspective that intersects *action-theoretical power* and *systemic power*, co-producers’ power to decentralise decision-making power was exercised through covert or explicit acts of resistance. In both cases, we observe that resistance decreases farm owners’ perceived or actual decision-making power, resulting in the increased power of co-producers and employees.

### Unmaking the monetisation of work for making non-monetised work relations

By creating the CSA, farm owners diversified the work relations on the farm and attributed a higher appreciation to non-monetised work relations. Three examples of alternative and non-monetised work relations that were highly appreciated in and across the three cases are worth describing. Firstly, in the case of CSA3, voluntary work by co-producers became increasingly essential to compensate for the uneven division of physical efforts and logistical work among farm owners and co-producers. When farm owners expressed their desire for summer holidays in 2020 and 2021, a group of co-producers self-organised a farm stay to allow farm owners to take holidays and keep the CSA operations going. Secondly, in the three CSAs, farm owners and co-producers often articulated the importance of permanent and temporary forms of voluntary work to decrease the operational costs of the CSA and secure a dignified income for farm owners and farm workers. Lastly, CSA members often highlighted the sociability character unique to their community interactions. Sociability, in practice, refers to the interactions based on care among co-producers, farm owners, and farm workers. Careful interactions also extended to the relation between CSA members and the farms’ ecosystem, according to one co-producer of CSA3:

As long as we don’t go there to exchange work for money, that’s a fundamental change that has repercussions on everything else. And actually, we’re going there to restore a bit of life as it is, and life implies social relationships. It implies a synergetic relationship with the land, food, and production. (co-producer, CSA3)

Although the creation of the three CSAs led to diversification of work relations in the farm and farm operations, CSAs did not eliminate waged work. Regarding the determination of wages, CSA2 and CSA3 farm owners’ salaries were discussed and agreed upon through collective processes. Conversely, in the case of CSA1, the farm owner tried to collectivise the decision of wages with the creation of the workers cooperative in 2018; however, the cooperative faced participation issues, and the farm owner continued determining wages alone. Additionally, the creation of CSA1 led to an increasing professionalisation of farm and CSA operations, as shown by the increase of salaried employees hired and the substitution of temporary volunteers by long-term interns from universities.

We identified different processes of unmaking capitalism in the attempt to value non-monetised work relations across the three cases (Table 5). We discuss them in turn.

**Table 5:** Evidence of processes of unmaking in the making of non-monetary work across the three CSAs

Non-monetary work relations	Process of unmaking	Case		
		CSA1	CSA2	CSA3
Voluntary work to even out physical efforts and logistical work among farm owners and co-producers	Sacrifice	–	–	Co-producers let go of certainty and expertise to run the farm while farm owners go on holidays
Voluntary work to decrease the operational costs of the CSA to secure a dignified income for farm owners and farm workers	Refusal	Co-producers refuse monetary compensation for their work	Co-producers refuse monetary compensation for their work	Co-producers refuse monetary compensation for their work
Work relations that attribute higher appreciation to sociability	Unlearning	Co-producers unlearn the logic of monetary compensation	Farm owners and co-producers discard the hierarchal interactions between producers and consumers	Farm owners and co-producers discard the hierarchal interactions between producers and consumers

**Sacrifice:** A group of CSA3 co-producers with limited farming experience let go of the need for certainty and agriculture expertise to voluntarily run the CSA farm while farm owners went on vacation. One co-producer explained:

I had little experience with farming. I didn’t know much about it. Sometimes we [with the farm owners] spoke about horticulture. Maybe I relied on this little confidence, like “If I were to be alone on the farm, I wouldn’t kill all the tomato plants”. (co-producer, CSA3)

Additionally, another co-producer of CSA3 explained that their individual choice to run the farm was part of a collective effort. Co-producers, volunteers, and one employee gathered to organise the farm stay, allowing them to experience less responsibility pressure:

I didn’t really feel the weight of responsibility… I didn’t know much about agriculture, but [the farm owners] explained what to do, and [the volunteer] knew what needed to be done for watering the fields. The employee also came in the mornings to organise. (co-producer, CSA3)

Through a *systemic power* perspective, CSA3 created internal social norms that prioritised the collectivisation of individual needs. Co-producers needed to sacrifice to pursue the collective responsibility of farm owners’ well-being. Although sacrifice was a personal choice, it was a joint effort that, in turn, helped alleviate responsibility pressure. Additionally, the collective organisation of the farm owners’ vacations helped allocate higher value to voluntary work. It provided co-producers with non-monetised outcomes in the forms of fulfilment and solidarity to enable others to enjoy rest and amusement.

**Refusal:** Refusal refers to the individual rejection of some affiliations to reconfigure social relations (Appendix A). Across the three cases, co-producers rejected the notion that work is legitimised only through monetary compensations to engage with and hold accountability over voluntary work for the CSA. In CSA2, co-producers who wrote the CSA newsletters, organised events, or set up administrative documents refused to be compensated for their working hours as they viewed voluntary work as necessary for the project’s viability. In the case of CSA3, co-producers realised that their financial contribution to the CSA resulted in low remunerations for the farm owners. During a CSA meeting organised to address this situation, co-producers refused a proposal to increase their financial contribution. Instead, co-producers re-articulated the value of voluntary work to compensate for the non-paid working hours of farm owners. One co-producer of CSA3 explained the implication of refusing monetary remuneration for the organisation of work in the CSA:

[The farm owners] are not properly paid for their work. Therefore, our participation in help-out gatherings and the distribution shifts must compensate for certain farm activities we don’t do. So, we pay in kind. We pay [the extra part of their remuneration] through our services. We pay one part financially and the other part through work. (co-producer, CSA3)

While money is an abstract form of compensation that allows workers to pursue their interests, community work prioritises the production of concrete collective benefits, for example, social bonds and knowledge sharing. Another CSA1 co-producer refused monetary compensation for their voluntary work as they prioritised the social results and the possibility of building knowledge through their engagement:

The help-out gatherings were proposed by us [co-producers] to foster our participation in the project. If I’m not mistaken, I think it was [the farm owner] who spoke many times about compensating people [...] When people proposed [the help-out gatherings], they proposed it to help, to help and to understand better how things are done [at the farm]. That’s it, without expecting anything in return. (co-producer, CSA1)

From an *action-theoretical* standpoint, refusing monetary compensation fostered a *reconfiguration of hierarchical work relations* that prioritised *collaborative work relations*. Asserting their voluntary intention to perform work for the community, co-producers stopped the reproduction of a relationship between the farm owner and worker in which the former is the one who solely benefits from the latter’s work. Instead, co-producers work for the community voluntarily because they also benefit from the dynamics and outcomes of communitarian work.

In *systemic power* terms, the refusal of monetary compensation for work performed for the CSA is entangled with class privilege. While the CSA enables the rearrangement of hierarchical work relations to prioritise collaborative ties between members, the CSA remains the primary source of income for farm owners to secure their livelihoods. Refusing monetary compensation for the work performed for the CSA was not equally manifested among CSA members. Non-monetised work in CSAs was possible only for those who had already secured their income elsewhere. Arguably, the refusal of monetary compensation may function as a diagnosis of socio-economic disparities and privilege within CSAs.

**Unlearning:** By engaging with the CSA, co-producers and farm owners questioned the taken-for-granted “service” mentality underlying conventional market-based interactions between producers and consumers to create relationships of physical and emotional proximity between all CSA members. Generally, the “service” mentality implies a hierarchical relation between producers and consumers. Consumers detain purchasing power and demand a type of service or product from food producers that meet their expectations in exchange for monetary payment. One co-producer of CSA2 explained how they discarded the “service” culture after joining the CSA:

I think that when people commit to the CSA they adopt a certain mentality. […]. There is empathy! Also, because of the type of relationships created [in the CSA]. In the city, we experience a distance [between producers and consumers], this mentality of: “I am paying. Therefore, I want to be served”. (co-producer, CSA2)

Similarly, a CSA3 co-producer pointed out that discarding the “service” mentality is a continuous conscious effort in CSAs, particularly in the case of new co-producers who have never participated in a CSA before:

[The CSA] is a completely revolutionary idea to acquire food. We have to repeat these ideas many times. It is not enough to say it in an assembly and write it in the minutes. It is the cultivation of this culture. Why? Because it goes against the idea of the market, which is you pay for the service, you pay for everything, and you won’t work anymore. And if you work, you get paid. (co-producer, CSA3)

Through the lens of *action-theoretical power*, discarding the “service” mentality may provide CSA members with new abilities and agency necessary to ensure non-alienated and active participation in the collective. Yet, such an unlearning experience can be contradictory. For example, CSA2 enabled a cheese producer to explore non-monetised work collaborations with co-producers. Although they valued the sociability aspect of collaborative work, they felt uncomfortable adopting a new role in the CSA. Meeting the expectations associated with consumers and work partners did not come naturally to them particularly because the relationship producer—co-producer included a monetary exchange (e.g., co-producers paid for her cheese) at the same time as a non-monetary collaboration (e.g., co-producers assisted in the logistics of ordering and distributing the cheese). At times, they felt uncomfortable negotiating their preferences for the logistics due to the persistent expectation of prioritizing the needs of co-producers, as they were the ones paying for the work.

### Unmaking the structural separation between productive and reproductive for making work relations full of care

In the capitalist organisation of work, reproductive work, such as care work to regenerate social and ecological lives, is understood as an (invisible) “maintenance basis” for productive work, such as food provisioning work performed to produce exchange value and generate an income (Dengler & Strunk 2018; Pungas 2020). Results indicate attempts by the selected CSAs to create agriculture and community practices that attributed visibility and recognition to care. We highlight two of these attempts and the aspects of the separation they aimed to reconcile. Firstly, CSAs articulated discourses and new language to deliberately recognise and valorise reproductive work. In CSA2, CSA members discussed their financial contribution beyond the payment for the productive work of farm owners and their reproductive work to regenerate the farm’s ecosystem.

Similarly, members of CSA3 proposed to name the vegetable basket “share” to shift the attention to the collective act of sharing the produce provided by the farm’s ecosystem. Also, the farm owner, employees, and co-producers of CSA1 explained that the CSA was conceived to shift farm operations from the market economy to a planned economy. Doing so enabled a farm organisation that operated following the rhythm of agroecological work, as explained by the farm owner:

CSAs are not an instrument of the conventional market; instead, they are a planned economy model. CSAs are closer to the temporality of agroecology than the market since agroecology encompasses long-term decisions, while the conventional market encompasses short-term decisions. (farm owner, CSA1)

Secondly, in CSA3, farm owners and co-producers explicitly organised reproductive tasks at distribution points and the farm. Parents organised child care and children’s activities among themselves during help-out gatherings and school vacations. Co-producers running the distribution point created a schedule to manage housekeeping tasks and foster rotating roles. Farm owners deliberately systematised housekeeping and cooking tasks on the farm to secure gender equality. Yet, results confirm previous findings that gender issues are not central to CSA debates yet shape everyday interactions and micro-politics (Homs et al. 2020). Despite some attempts to discuss unequal gender division of reproductive work in smaller groups, CSA3 co-producers commented that most reproductive tasks were mainly performed by women, and such an issue never became a prominent topic in the collective debates. These attempts to create work relations full of care in CSAs were influenced by the unmaking of different aspects of the structural separation between productive and reproductive work (Table 6).

**Table 6:** Evidence of processes of unmaking in making work relations full of care across the three CSAs

Work relations full of care	Process of unmaking	Case		
		CSA1	CSA2	CSA3
Discourses and new language to deliberately recognise and valorise reproductive work	Defamiliarisation	–	Disruption of common sense that co-producers pay only for the product	De-automatisation of the commercial meaning of a CSA basket
	Unlearning	–	Discard the belief that in a CSA, the farm infrastructure was the only responsibility of farm owners	–
The explicit organisation of reproductive tasks	Resistance and sacrifice	–	–	Resistance to the devaluation and invisibility of care work. Individual sacrifice to perform care work to benefit the group
	Resistance and refusal	–	–	Visible and invisible objection to the devaluation of care work and gender inequality

**Defamiliarisation:** Defamiliarisation refers to de-automatising an act or object by showing it in a novel or unusual light to make someone conscious of differences (Appendix A). Members of CSA2 and CSA3 engaged in collective activities that aimed to deliberately de-habituate their automatised perceptions of some of their CSA operations to generate visibility and higher valorisation of work performed to regenerate the farm’s ecosystem. In the case of CSA2, a group dynamic exercise organised during the assembly in July 2021 invited co-producers to indicate whether they paid for the products in the CSA basket or the work performed by farm owners to regenerate their farmland and be able to share the harvest with the co-producers. The group dynamic exercise intended to disrupt the common sense that co-producers paid only for the provisioning work and not the care work to regenerate the farm’s ecosystem.

Similarly, participants of the CSA3 assembly in October 2021 discussed the proposition to re-name the CSA basket from “basket” to “share” to de-automatise the commercial perception often attributed to a vegetable basket. Some co-producers contested the proposition, claiming the new name was an empty signifier. Nonetheless, the proposal triggered reflexivity. As explained by one CSA3 co-producer:

The basket, the share. More and more, I realise that it is a sample of the farm because that’s what you can collect on a given day, right? Which is a result of [the farm owners’] work, of all the co-producers and co-producers, to keep that land fertile and productive, and so on. (co-producer, CSA3)

From a *constitutive power* perspective, introducing a new name and meaning to the CSA basket triggered more profound reflexivity among co-producers about their perception of and interaction with the basket. Although co-producers contested the term “share”, the new name proposition allowed them to realise that their role as co-producers and users of the “share” constituted a broader commitment to the regeneration of the soil. In other words, the interaction with a food basket called “share” sheds new light on the practice of producing or acquiring food aimed at by the CSA.

**Unlearning:** During the CSA assembly in July 2020, one of the CSA2 farm owners discarded the belief that farmers were exclusively responsible for improvements in the farm infrastructure. This argument reinforced shared accountability for the maintenance of the farm. The other CSA2 farm owner explained the incident:

[During the CSA assembly] I said that we [farm owners] really wanted to have a greenhouse. Then, [the male farm owner] intervened and said: “We don’t want to have a greenhouse. We, the CSA, need to have a greenhouse to guarantee winter production!” Wow, what an insight! [...] After that, co-producers got involved in all the phases for the greenhouse construction: fundraising and budget estimation. (farm owner, CSA2)

By stressing that “we” did not mean the farm owners but rather the CSA as a whole, the farm owner displaced the market-based belief that farm owners alone are responsible for covering the expenses of agriculture work. Subsequently, as explained above by the CSA farm owner, farm owners and co-producers gathered to organise a crowdfunding campaign to construct a greenhouse at the farm.

This unlearning process enabled a stronger alliance between CSA members and farm infrastructure to generate human and ecosystem benefits in *constitutive power* terms. On the one hand, the greenhouse construction strengthened group cohesion, revealing individual abilities and capacities that were not yet collectivised. On the other hand, the greenhouse enabled greater variety of produce during the winter season and generated ecosystem resilience to cope with challenging weather conditions (e.g., winter frost).

**Resistance and sacrifice:** Members of CSA3 attempted to resist the reproduction of a work organisation that devaluated and invisibilised housekeeping tasks to create a greater sense of collective accountability for reproductive work. In the autumn of 2020, co-producers running a distribution point received a complaint from their hosting institution alleging poor maintenance of the place. During an internal meeting to address the issue, co-producers discussed housekeeping tasks and created a rotating schedule to make these tasks explicit and encourage collective accountability.

On the one hand, the discussion helped create a greater sense of care for the location of the distribution point, as pointed out by one co-producer:

At the beginning, we were not very careful. In comparison to how it is now when we put an effort in cleaning tasks, the [distribution point] is very tight every week. (co-producer, CSA3)

On the other hand, the care for the location did not expand to the whole group. As explained by the same co-producer, the rotating schedule did not succeed, and housekeeping tasks continued to be performed by the usual suspects. According to them, one possible reason is the fact that these tasks are not paid:

Lately, we have discussed that the same people usually perform these tasks. And there are [schedule] sheets. These sheets were made for this purpose [encourage rotating tasks]. But maybe it is because these tasks are not paid…Well, we have never talked about it… But yes, in fact, that could be a reason. (co-producer, CSA3)

The rotating schedule failed to resist a devaluation and invisibility of housekeeping tasks, and a careful relationship with the space remained limited to a few co-producers. Some of these co-producers, in turn, commented that they had to sacrifice their individual preferences to benefit the whole group.

When I arrive at the [distribution point], I check what is needed to do and how to contribute to logistic tasks, like locking [the doors of the distribution point] and cleaning. This has been an issue since the beginning. These tasks are not explicit for everyone, also as rotating tasks. I don’t necessarily like to take on these tasks every week, but it ends up being like this. But this is obvious, right? This is about self-management. We need to organise. (co-producer, CSA3)

From a *constitutive power* perspective, the rotating schedule enabled more visibility to care work; however, it did not constitute sufficient hindrance against the devaluation of care work. The rotating schedule empowered co-producers to systematise housekeeping activities in the distribution point but insufficiently disrupted a devaluation of care work more deeply ingrained in the local culture that, among other possible reasons, attributes more value to traditionally paid work than to traditionally unpaid work, such as housekeeping.

**Resistance and refusal:** The female farm owner of CSA3 objected to the devaluation and invisibility of her housekeeping, cooking, and farm work to ensure a just distribution of care and provisioning tasks that preserved gender equality. Objections happened through covert and overt acts.

Currently, the male farm owner is in charge of farming for the CSA, and the female farm owner is responsible for administrative tasks for the CSA. When asked how such a division came to be, both farm owners answered that it was a natural process. However, each had a different view on how gender norms shaped the organisation of tasks. For the male farm owner, he took on farming activities because, as a father, he was the one available for the job. The female farm owner, instead, was available for administrative work as her motherhood duties prevented her from doing farm work:

The tasks of a mother with a newborn child ended up draining a lot of energy from her that would be necessary to work in the field, tilling, planting, etc. And this turns out to be a job for the father because he is available. […] Besides, I was tired, and the last thing I wanted to do was to be held on the phone or the computer. [*laughs*]. [The female farm owner], on the other hand, although she didn’t like it very much either, because she also wanted to be in the garden, ended up being the one available [to perform administrative work]. (male farm owner, CSA3)

The female farm owner implicitly objected to the devaluation of her farm work by the male partner by refusing to perform some farm work she did not feel valorised to do:

He does some of the farm work that I don’t do. I don’t know how to do it. And I decided that I didn’t want to know, either. For instance, watering plants requires a lot of work. I don’t care [..] I already have a lot of other things to do. He does it, and if you want to do it too, or to learn how to do it: cool! I don’t want to. (female farm owner, CSA3)

Moreover, the female farm owner pointed out the influence of gender norms on the uneven distribution of value across the work she and her partner do. She explicitly objected to her partner’s devaluation of housekeeping and cooking tasks by re-arranging responsibilities and holding him accountable for some of these tasks:

It is a gender issue, and I won’t lie. […] Because there is also this thing that sometimes some work is not as recognised as it should be […] Because there were these moments, “I do this, and this, and this all. Therefore, I cannot cook” [referring to her partner][…] Now we have organised these tasks. I and the others that come here [at the communal kitchen] cook. He does the dishes in our house. I do the dishes here. We have been fine-tuning after so many discussions about this issue. Now we have found a balance. (female farm owner, CSA3)

From a *systemic power* perspective, gender norms influenced the *uneven value distribution* to provision and care work. The farm owners embodied the expectations of motherhood and fatherhood duties when distributing work among themselves. Such a distribution originated when their child was born and had an enduring effect on the organisation of farm work and CSA responsibilities. In *action-theoretical power* terms, the female farm owner exercised *invisible and visible power* to object to the unjust patterns of such distribution. Arguably, her invisible objection may have enabled her to self-affirm her role on the farm despite the level of valorisation conceived by her partner. But also, such objection resulted in a coping mechanism to deal with a devaluation of her farm work deeply ingrained in her partner’s perception of gender division of farm work.

## Discussion and conclusion

### Power in transformations towards postcapitalist work relations in CSAs

Our study analysed the role of power in transformations towards postcapitalist work relations in three CSAs. We looked at postcapitalist transformations as a political process of diversification that reattributes value to alternative- and non-capitalist work relations traditionally undervalued and invisibilised (Gibson-Graham 2006, 2008). We combined three theorisations of power (action-theoretical, constitutive, and systemic) (Allen 2021) with an approach to transformations as processes of unmaking capitalist relationships and practices to make space for postcapitalist alternatives (Feola 2019; Feola et al. 2021) (Table 1). We used this conceptual framework to analyse how power enabled or constrained the transformation of three aspects of work relations—alienation, monetisation, and care—based on empirical evidence from three CSAs in Portugal.

This paper makes two significant contributions to research on CSA and similar agri-food grassroots initiatives pursuing transformations to sustainability beyond capitalism. Firstly, it tackles the lack of research on the processes through which postcapitalist transformations unfold by identifying and examining processes of unlearning, sacrifice, resistance and everyday resistance, defamiliarisation, and refusal that pre-condition the making of postcapitalist work relations in CSAs (Tables 4, 5, and 6). Secondly, our study addressed recent calls for further analyses of power in postcapitalist transformations (Gabriel & Sarmiento 2020; Wilson & Mutersbaugh 2020; Turker & Murphy 2021; Morrow & Davies 2022) by offering new insights into action-theoretical, constitutive, and systemic manifestations of power shaping instances of (un)making in transformations to postcapitalist work relations (Table 3).

Notwithstanding the relatively limited sample, this study offers valuable insights about transformations in CSAs in practice. A critical finding of our analysis is that the three CSAs analysed created diverse work relations among co-producers, employees, and farm owners, as previously discussed in the literature (e.g., Thompson & Coskuner-Balli 2007; Wilson 2013; Ekers 2019; Watson 2020); yet, the reattribution of value to alternative- and non-capitalist work relations is uncertain, and these CSAs reconfigure only to a limited extent the hierarchal, exploitative, and discriminatory relations that characterise capitalist work relations (e.g., Duffy 2007; Dengler & Strunk 2018). In particular, two approaches to diversifying work relations in CSA emerge from this study. First, the three CSAs implemented participatory mechanisms, such as sociocracy, to structure the distribution of tasks and responsibilities and to negotiate the reattribution of value to work activities traditionally obscured within capitalism. Second, farm owners encouraged meaningful and enjoyable work relations through synergetic human–non-human interactions as noticed in the co-construction of the farm infrastructure to enhance participation of members and collective accountability over CSA operations in CSAs 2 and 3, and through attempts to re-signify the interactions between farming work, co-producers, and the CSA basket in CSA3. We have shown that these approaches partially helped the selected CSAs achieve their envisioned postcapitalist work relations. While these CSAs focused on creating solutions to enable postcapitalist work relations that are non-alienated, non-monetised, and full of care, they insufficiently unmade unbalanced power relations established in capitalist work relations.

We highlight two unbalanced power relations reproduced in the selected case studies that constrained transformations to postcapitalist work relations. On the one hand, the selected CSAs were founded and led by farm owners, and their leading role reproduced hierarchal ties among them, co-producers, and employees. Such hierarchal relations created difficulties for maintaining non-alienated work relations. In contrast to Watson (2020), who argued that democratic governance structures implemented by CSAs enable all members to influence decisions and define work, and White (2013), who stressed that work arrangement between farm owners and volunteer workers favour autonomy, our results showed that the leading role of farm owners in all three CSAs centralised abilities and knowledge on them and hindered the participation of co-producers and employees in decision-making meetings and the arrangement of tasks and responsibilities. On the other hand, collaborative interactions among farm owners, employees, and co-producers to decide and execute CSA operations were limited by the historical and situated constitution of uneven power relations, as also discussed by, for example, Galt (2013) and Ekers (2019). In CSA1, the participation of employees and co-producers in decision-making or unpaid activities of the CSA was scarce due to their region’s traditional boss–worker hierarchal culture. Similarly to Sbicca (2015) and Levkoe and Offeh-Gyimah (2020), while farm owners and co-producers of CSA 2 and 3 sacrificed their leisure time to work for the CSA, their sacrifice motives differed and, in the case of co-producers, sacrifice was entangled with class privilege. In CSA3, the invisibility of care work in capitalist systems, as pointed out by Dengler and Strunk (2018), hindered co-producers’ further accountability for maintaining their distribution point. Also, gender norms influenced an enduring devaluation and uneven distribution of care work between the male and female farm owners, as discussed by Wells and Gradwell (2001).

### Implications for studies on agri-food grassroots initiatives for transformations to sustainability

We propose two implications for the scholarship on agri-food grassroots initiatives for postcapitalist transformations of agri-food systems, including studies of CSAs. Firstly, we observed that across the three cases, the power to *decentralise* tasks and responsibilities and to involve members in CSA operations became increasingly *centralised* on farm owners. The centralisation of decision-making power on farm owners reinforced hierarchal relations in all of the three CSAs. Subsequently, co-producers and employees relied on farm owners’ coordination to participate in the initiative instead of feeling empowered to autonomously support or contest farm owners’ decisions and actively shape the distribution of tasks and responsibilities across CSA members. This case is similar to the paradox of empowerment put forth by political scientists (Hardy & Leiba-O’Sullivan 1998; Avelino 2011; Schreuer 2016). Schreuer (2016) explains that *“*the notion of one actor empowering another through the provision of particular resources is inherently paradoxical, as this makes the supposedly empowered actor newly dependent on this channel of resource” (p. 134). While some CSA members appreciated the coordination role of farm owners, the cases of CSA1 and CSA3 illustrate visible and invisible attempts of co-producers to resist and diminish the power of farm owners. Conversely, unlearning hierarchal relations between CSA members can be a contradictory personal experience (see Feola 2019; van Oers et al. 2023), as illustrated by the case of one associated producer of CSA2. These findings highlight some of the barriers and opportunities faced by the three Portuguese CSAs for decentralising power relations and suggest that in order to fully accomplish transformations towards postcapitalist work relations, these initiatives may benefit from implementing horizontal and participatory mechanisms and actively deconstructing internal hierarchies and the centralisation of power.

Secondly, and in relation to the previous point, the selected CSAs showcase how the internal negotiations for a just and meaningful attribution of value to different forms of work relations in CSAs are strongly influenced by power relations established by structures of oppression. Our findings showed that collaborative interactions among farm owners, employees, and co-producers to decide and execute CSA operations could be limited because of the historical and situated constitution of uneven power relations. For instance, the case of CSA1 illustrates how participatory decision-making mechanisms aimed to resist and overcome hierarchical work relations are constrained by a traditional boss–worker culture embodied by employees. Since the beginning of the twentieth century, large agricultural estates have prevailed in the Alentejo region, where CSA1 is located, due to a state-led programme to modernise the agricultural sector (Calvário 2022). The modernisation of agriculture in Alentejo was characterised by little mechanisation of farms and heavy dependence on long-term waged workers, resulting in the growing proletarianisation of the rural population (do Carmo 2010). Also, the illiteracy rates of the rural working class remained high (Russo de 2014). Such a political conjecture historically allocated more power to land owners than farm workers and consolidated hierarchical work relations. Therefore, we contend that future research on agri-food grassroots initiatives must seriously consider and actively address oppressive power relations that are ossified in the local and cultural context where these initiatives are situated and influence the implementation of participatory and horizontal decision-making mechanisms.

To conclude, we encourage future research on the role of power in tensions between deconstruction and construction in CSAs that embrace the *gender* dimension. Our analysis of transformations towards postcapitalist work relations revealed how gender norms shaped the internal organisation of work and influenced the uneven attribution of value to care and provisioning work to male and female farm owners. Arguably, these results remained limited to the case of unmaking the structural separation between reproductive and productive work because the literature we referenced on this topic offers several critiques of capitalist organisations of work and their implications for the reproduction of gender (in)equality (Duffy 2007; Pungas 2020). Although the gender dimension is of particular interest to the case of reproductive and productive work, this dimension is not exclusive to this case. Gender studies and feminist analyses of CSAs have discussed how these initiatives create social spaces for women’s self-identification and reproductive roles, including community building (e.g., Cone & Myhre 2000; Wells & Gradwell 2001; Jarosz 2011). Future studies on power in CSAs can benefit from deeper engagement with the gender dimension, for instance, to analyse how individual trajectories of becoming a male, female, or queer farmer shape the tensions between deconstruction and construction within collective processes of transformations.

## Appendix A

Inventory of theories of Unmaking capitalism (Feola et al. 2021).

**Table:**

Theory/concept (field)	Selected references	Core Idea	Levels at which it occurs	Significance for the unmaking of capitalist work relations*	Significance for the making of postcapitalist work relations*
Destabilisation (Sustainability transitions)	Turnheim and Geels (2013)	The ‘process of weakening reproduction of core [sociotechnical] regime elements’ such as routines, technical capabilities, strategic orientation, and mindsets (Turnheim and Geels, 2012, p. 35)	Macro (societal)	Weakens the reproduction of core elements of capitalist socio-technical regimes (e.g., technical capabilities for the increasing exploitation of human and non-human life, strategic orientation towards efficiency)	Allows cultural, technical and strategic diversification and experimentation (e.g., as related to modes of exchange outside of the market, responsible technologies or strategic orientation towards sufficiency)
Exnovation (Sustainability transitions)	Davidson (2019)	A ‘conscious decision to phase out technology or practice, to decommission it, and to withdraw the corresponding resources and use them for other purposes’ (Kimberly 1981, p. 91)	Macro (societal)	Abandons, purposively terminates, defunds, deroutinizes and, or deinstitutionalises socially and environmentally destructive or exploitative technologies, and the production and consumption practices with which they are bound	Allows political and financial capital to be invested in alternative technologies (e.g., low-tech, frugal technologies) and related practices, value systems (e.g., oriented towards care), and more horizontal power structures
Unlearning (Organisation studies)	(Fiol and O’Connor, 2017a, 2017b)	Consciously not thinking or acting in ‘old’ ways (Stenvall et al., 2018)	Micro (individual), Meso (collectives)	Abandons, rejects, discards from use, gives up, abstains from retrieving, questions taken-for-granted values, norms, beliefs (e.g., the idea of progress as endless accumulation and expansion), and operations and behaviour (e.g., over-production and consumption)	Enables learning new cultural significations and routines (e.g., voluntary simplicity) and emotional re-attachment (e.g., with nature)
Sacrifice (Political ecology)	Maniates and Meyer (2010)	Giving up something (now) for something of higher value (to be obtained now or in the future)	Micro (individual), Meso (collectives)	Entails voluntary reduction of consumption (voluntary simplicity)	Enables time and space for developing new cultural significations and practices, e.g., as related to non-utilitarian, non-market-based engagements with the self, others, and the biophysical environment
Crack capitalism (Social movement studies and autonomous geographies)	Holloway (2010)	A refusal to perpetuate capitalist practices and organisational structures through its commitment to value, money, profit	Micro (individual), Meso (collectives)	Entails the refusal to reproduce capitalist relations (e.g. labour, value). Rejects rigid classifications and totalising abstractions (value, labour) as expressions of modern rationalism and capitalist form of domination	Enables autonomy to enact forms of doing and organising based on nonmonetary values, self-determination, horizontal relations, and principles of cooperation and recognition
Everyday resistance (Peasant and development studies)	Scott (1986)	Everyday resistance refers to quiet, dispersed, disguised, or otherwise seemingly invisible acts of opposition, struggle or refusal to cooperate with abusive powers	Micro (individual), Meso (collectives)	Questions, opposes and objects to abusive or oppressive power relations. Refuses to cooperate with or submit to oppressive behaviour and control (e.g., as it relates to the appropriation and exploitation of cheap nature and labour)	Enables autonomy and sense of dignity
Resistance (Social movement and political studies)	Hollander and Einwohner (2004)	Resistance refers to varying forms of overt (visible) intentional actions of opposition, which are recognised by the targets of such opposition	Meso (collectives), Macro (societal)	Questions, opposes and objects to abusive or oppressive power relations. Actively dismantles material and symbolic infrastructures of capitalist exploitation of human or non-human life; contests and prevents the physical or symbolic presence of organisations imposing capitalist institutions and relations	Defends spaces of diversity and autonomy. Reinforces alternative identities through collective action
Refusal (Decolonial and cultural studies)	McGranahan (2016) Simpson (2016)	Refusal is the rejection or negation of an imposed and taken-for-granted definition of a situation, identity and/or social relation	Micro (individual), Meso (collectives)	Abstains from, stops, and/or breaks exploitative and/or alienating relations (e.g. labour relations). Rejects (taken for granted) consent to, e.g., definitions of progress as endless accumulation or consumption as only political space	Affirms freedom to redefine identities, problem definitions, histories; thereby provides alternative basis for social recognition, empowerment and reconfiguration of social relations on the ground of, e.g., principles of care, democracy, autonomy
Delinking (Decolonial and cultural studies)	Mignolo (2007) Wazner-Serrano (2015)	De-linking from the colonial rhetoric of modernity, which must be conceived as simultaneously capitalist, and denouncing the pretended universality of a Western and European episteme in which capital accumulated as a consequence of colonialism	Meso (collectives), Macro (societal)	Uncovers hidden assumptions, rejects/resists claims to epistemic privilege and universality of Western thought. Disengages from the logic and rhetoric of modernity and capitalism	Allows claiming and relinking with diverse (e.g., relational) logics and types of knowledge (e.g., non-scientific) and a redefinition of citizenship, democracy, human rights, human and non-human nature, economic relations
Decolonisation of the imaginary (Degrowth)	Latouche (2010)	A radical and profound cultural change of the foundational imaginary significations of modern capitalist societies	Micro (individual), Meso (collectives), Macro (societal)	Refuses complicity and collaboration with the ideology of development, e.g., as in the abstention from the use of environmentally destructive technologies, or the limitation of space allotted for advertisement. Cognitively subverts and critiques economicism and the imperative of endless economic growth	Enables the autonomous determination of new imaginaries (e.g., alternatives to development)
Defamiliarization (Decolonial and cultural studies)	Shklovsky (1925) Vaught (2012)	The ‘removal of an object of the sphere of automatised perception’ (Shklovsky, 1925, p.6)	Micro (individual)	Ruptures, de-automatises, dishabituates automised perception, e.g., as related to cultural constructions of value and worth. Emotional detachment and critical reflection. Disrupts common sense, e.g., as related to taken-for-granted production-consumption routines and utilitarian value systems	Allows critical awareness, emotional re-attachment, and establishment of new cultural meanings

*We provide here an interpretation (stretching) of the theories and concepts to illustrate their applicability to and significance for the study of the unmaking of capitalist modernity and the making of post-capitalist realities. The examples are illustrative and not comprehensive.

## Abbreviations

CSA: Community-supported agriculture
CSAs: Community-supported agriculture initiatives

## Footnotes

1 We follow Gibson-Graham’s (2006) definitions of capitalist, alternative- and non-capitalist work. Capitalist work relations include paid labour in which employers and employees (or worker unions) negotiate the terms and conditions (e.g., salary, benefits, power relations) of the employment contract. Alternative-capitalist work relations, in turn, refer to labour that is paid differently than capitalist wages in the forms of collectively or individually defined living wages (e.g., cooperative salary) and payment-in-kind (e.g., labour in exchange for food and shelter). Non-capitalist work relations are unremunerated in monetary terms. Instead, they are compensated by affection (e.g., emotional support) or subsistence (e.g., food), and workers can directly enjoy the outcomes of their work (e.g., meals for themselves and their families). Conversely, non-capitalist work relations also encompass enslaved labour that is unpaid and unfree (e.g., sex slavery).

2 The selected CSAs adopted the term “co-producer” as an alternative to the term “consumer”. Generally, the new term intended to spur active participation and shared accountability over the economic viability and labour for agri-food production, in contrast to the passive role of consumers performed in conventional market transactions. Nonetheless, this behaviour and mindset shift remained a challenge for most of the CSAs, as the work share of co-producers was significantly smaller than the work performed by farm owners and employees, and their involvement in work tasks was optional.

## References

[CR1] Ahlborg H (2017). Towards a conceptualization of power in energy transitions. Environmental Innovation and Societal Transitions.

[CR2] Allen, A. 2021. *Stanford encyclopedia of philosophy feminist perspectives on sex and gender*, 1–23.

[CR3] Avelino F (2011). Power in transition: Empowering discourses on sustainability transitions. Erasmus Universiteit Rotterdam, Rotterdam..

[CR4] Calvário R (2022). The making of peasant subalternity in Portugal: Histories of marginalisation and resistance to agrarian modernisation. The Journal of Peasant Studies.

[CR5] Cone CA, Myhre A (2000). Community-supported agriculture: A sustainable alternative to industrial agriculture?. Human Organization.

[CR6] Cristiano S (2021). Organic vegetables from community-supported agriculture in Italy: Emergy assessment and potential for sustainable, just, and resilient urban-rural local food production. Journal of Cleaner Production.

[CR7] de Russo RHO (2014). Os Trabalhadores Rurais em Portugal nas Primeiras Décadas do Século XX (1900–1920): Perspectivas Historiográficas. Anais Eletrônicos Do XXII Encontro Estadual De História Da ANPUH-SP.

[CR8] Delind LB, Ferguson AE (1999). Is this a women ft movement: The relationship of gender to community-supported agriculture in Michigan. Human Organization.

[CR9] Dengler C, Strunk B (2018). The monetized economy versus care and the environment: Degrowth perspectives on reconciling an antagonism. Feminist Economics.

[CR10] do Carmo, R. M.  (2010). A agricultura familiar em Portugal: Rupturas e continuidades. Revista De Economia e Sociologia Rural.

[CR11] Duffy M (2007). Doing the dirty work. Gender, Race, and Reproductive Labor in Historical Perspective..

[CR12] Ekers M (2019). The curious case of ecological farm interns: On the populism and political economy of agro-ecological farm work. Journal of Peasant Studies.

[CR13] Ekers M, Levkoe CZ, Walker S, Dale B (2016). Will work for food: Agricultural interns, apprentices, volunteers, and the agrarian question. Agriculture and Human Values.

[CR14] Esteves AM (2017). “Commoning” at the borderland: Ecovillage development, socio-economic segregation and institutional mediation in southwestern Alentejo, Portugal. Journal of Political Ecology.

[CR15] Feagan R, Henderson A (2009). Devon acres CSA: Local struggles in a global food system. Agriculture and Human Values.

[CR16] Feola G (2019). Degrowth and the unmaking of capitalism: Beyond ‘decolonization of the imaginary”. Acme.

[CR17] Feola G (2020). Capitalism in sustainability transitions research: Time for a critical turn?. Environmental Innovation and Societal Transitions.

[CR18] Feola G, Vincent O, Moore D (2021). (Un)making in sustainability transformation beyond capitalism. Global Environmental Change.

[CR19] Gabriel N, Sarmiento E (2020). Troubling power: An introduction to a special issue on power in community economies. Rethinking Marxism.

[CR20] Galt RE (2013). The moral economy is a double-edged sword: Explaining farmers’ earnings and self-exploitation in community-supported agriculture. Economic Geography.

[CR21] Galt RE, Bradley K, Christensen LO, Munden-Dixon K (2019). The (un)making of “CSA people”: Member retention and the customization paradox in community supported agriculture (CSA) in California. Journal of Rural Studies.

[CR22] Gibson-Graham JK (2006). A postcapitalist politics.

[CR23] Gibson-Graham JK (2008). Diverse economies: Performative practices forother worlds’. Progress in Human Geography.

[CR24] Hardy C, Leiba-O’Sullivan S (1998). The power behind empowerment: Implications for research and practice. Human Relations.

[CR25] Harris E (2009). Neoliberal subjectivities or a politics of the possible? Reading for difference in alternative food networks. Area.

[CR26] Homs P, Flores-Pons G, Mayor AM (2020). Sustaining caring livelihoods: Agroecological cooperativism in Catalonia. Food for Degrowth.

[CR27] INE (Instituto Nacional de Estatística) (2021). Recenseamento Agrícola, Análise dos principais resulta-dos: 2019.

[CR28] INE (Instituto Nacional de Estatística).  (2022). XVI Recenseamento Geral da População: VI Rescenseamento Geral da Habitação, Resultados definitivos.

[CR29] Jarosz L (2011). Nourishing women: Toward a feminist political ecology of community supported agriculture in the United States. Gender, Place and Culture.

[CR30] Levkoe CZ, Offeh-Gyimah A (2020). Race, privilege and the exclusivity of farm internships: Ecological agricultural education and the implications for food movements. Environment and Planning e: Nature and Space.

[CR31] Marx K (1959). Economic and philosophic manuscripts of 1844. Economica.

[CR32] Minkoff-Zern LA (2017). The case for taking account of labor in sustainable food systems in the United States. Renewable Agriculture and Food Systems.

[CR33] Moragues-Faus A, Marsden T, Adlerová B, Hausmanová T (2020). Building diverse, distributive, and territorialized agrifood economies to deliver sustainability and food security. Economic Geography.

[CR34] Morrow O, Davies A (2022). Creating careful circularities: Community composting in New York city. Transactions of the Institute of British Geographers.

[CR35] Myers JS, Sbicca J (2015). Bridging good food and good jobs: From secession to confrontation within alternative food movement politics. Geoforum.

[CR36] Nost E (2014). Scaling-up local foods: Commodity practice in community supported agriculture (CSA). Journal of Rural Studies.

[CR37] Novikova M (2021). Transformative social innovation in rural areas: Insights from a rural development initiative in the Portuguese region of Baixo Alentejo. European Countryside.

[CR38] Pereira C, Pereira A, Budal A, Dahal S, Daniel-Wrabetz J, Meshelemiah J, Carvalho J, Ramos MJ, Carmo RM, Pires RP (2021). ‘If you don’t migrate, you’re a nobody’: Migration recruitment networks and experiences of Nepalese farm workers in Portugal. Journal of Rural Studies.

[CR39] Pole A, Gray M (2013). Farming alone? What’s up with the “C” in community supported agriculture. Agriculture and Human Values.

[CR40] Pungas L (2020). Caring dachas: Food self-provisioning in Eastern Europe through the lens of care. Food for Degrowth.

[CR41] Raj G, Feola G, Hajer M, Runhaar H (2022). Power and empowerment of grassroots innovations for sustainability transitions: A review. Environmental Innovation and Societal Transitions.

[CR42] Rosol M (2020). On the significance of alternative economic practices: Reconceptualizing alterity in alternative food networks. Economic Geography.

[CR43] Sbicca J (2015). Food labor, economic inequality, and the imperfect politics of process in the alternative food movement. Agriculture and Human Values.

[CR44] Schreuer A (2016). The establishment of citizen power plants in Austria: A process of empowerment?. Energy Research and Social Science.

[CR45] Sharp EL, Petersen I, Mclellan G, Cavadino A, Lewis N (2022). Diverse values of surplus for a community economy of fish (eries). Asia Pacific Viewpoint.

[CR46] Thompson CJ, Coskuner-Balli G (2007). Enchanting ethical consumerism: The case of community supported agriculture. Journal of Consumer Culture.

[CR47] Trauger A, Passidomo C (2012). Towards a post-capitalist-politics of food: Cultivating subjects of community economies. Acme.

[CR48] Turker KA, Murphy JT (2021). Assembling community economies. Progress in Human Geography.

[CR49] van Oers L, Feola G, Runhaar H, Moors E (2023). Unlearning in sustainability transitions: Insight from two Dutch community-supported agriculture farms. Environmental Innovation and Societal Transitions.

[CR50] Vincent O, Feola G (2020). A framework for recognizing diversity beyond capitalism in agri-food systems. Journal of Rural Studies.

[CR51] Watson DJ (2020). Working the fields: The organization of labour in community supported agriculture. Organization.

[CR52] Weiler AM, Otero G, Wittman H (2016). Rock stars and bad apples: Moral economies of alternative food networks and precarious farm work regimes. Antipode.

[CR53] Wells BL, Gradwell S (2001). Gender and resource management: Community supported agriculture as caring-practice. Agriculture and Human Values.

[CR54] White T (2013). Growing diverse economies through community supported agriculture. Northeastern Geographer.

[CR55] Wilson AD (2013). Beyond alternative: Exploring the potential for autonomous food spaces. Antipode.

[CR56] Wilson BR, Mutersbaugh T (2020). Solidarity interrupted: Coffee, cooperatives, and certification conflicts in Mexico and Nicaragua. Rethinking Marxism.

